# Simultaneous acquisition of current and lateral force signals during AFM for characterising the piezoelectric and triboelectric effects of ZnO nanorods

**DOI:** 10.1038/s41598-021-82506-8

**Published:** 2021-02-03

**Authors:** Yijun Yang, Kwanlae Kim

**Affiliations:** Department of Manufacturing Systems and Design Engineering (MSDE), Seoul National University of Science and Technology (SeoulTech), Seoul, 01811 Republic of Korea

**Keywords:** Energy science and technology, Materials science, Nanoscience and technology, Physics

## Abstract

Atomic force microscopy (AFM) is central to investigating the piezoelectric potentials of one-dimensional nanomaterials. The AFM probe is used to deflect individual piezoelectric nanorods and to measure the resultant current. However, the torsion data of AFM probes have not been exploited to elucidate the relationship between the applied mechanical force and resultant current. In this study, the effect of the size of ZnO nanorods on the efficiency of conversion of the applied mechanical force into current was investigated by simultaneously acquiring the conductive AFM and lateral force microscopy signals. The conversion efficiency was calculated based on linear regression analysis of the scatter plot of the data. This method is suitable for determining the conversion efficiencies of all types of freestanding piezoelectric nanomaterials grown under different conditions. A pixel-wise comparison of the current and lateral force images elucidated the mechanism of current generation from dense arrays of ZnO nanorods. The current signals generated from the ZnO nanorods by the AFM probe originated from the piezoelectric and triboelectric effects. The current signals contributed by the triboelectric effect were alleviated by using an AFM probe with a smaller spring constant and reducing the normal force.

## Introduction

**Figure 1:**
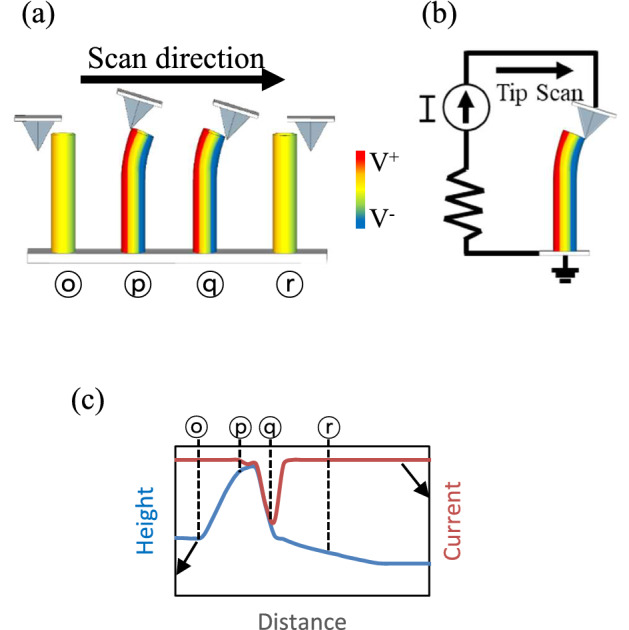
Piezoelectric potential induced by the AFM probe. (**a**) Generation of piezoelectric potential by deflection of the ZnO nanorod, (**b**) current passing through the metal-coated AFM tip in contact with the ZnO nanorod, (**c**) topography and C-AFM signals during the process in (**a**). The current signals originating from the piezoelectric potential are detected when the AFM tip touches the compressed side of an n-type ZnO nanorod^1,9^.

The piezoelectric effect of ZnO nanorods was first reported by Wang and Song in 2006^1^. Subsequently, an atomic force microscopy (AFM)^2,3^ tip was used to deflect vertically grown ZnO nanorods at the submicron scale. Because ZnO exhibits semiconducting and piezoelectric properties^4–6^, the strain induced in the individual ZnO nanorods by the AFM tip drives a flow of electric charge carriers through the metal-coated AFM tip and ZnO nanorods. Furthermore, the correlation between the topography signal and conductive atomic force microscopy (C-AFM)^7,8^ signal was analyzed to elucidate the underlying mechanism responsible for the generation of a piezoelectric potential in ZnO nanorods and to detect the current signal via the AFM tip. Through this experimental study, it was shown that the current signal during C-AFM is detected when the AFM tip touches the compressed side of an *n*-type ZnO nanorod^9^, as presented in Fig. 1. This comparative analysis of the topography and C-AFM signals also helped elucidate the piezoelectric effect in *p*-type ZnO nanorods^10^ and improved understanding of the effects of the ZnO nanorod growth method used on the piezoelectric power generation characteristics of the resulting nanorods^11^. C-AFM is used preferentially to investigate the piezoelectricity of one-dimensional nanomaterials with wurtzite structures, such as CdS^12^, CdSe^13^, ZnS^14^, InN^15^, and GaN^16^.

Various types of sensors and energy harvesters based on piezoelectric nanomaterials and with a range of structures have been investigated^17–22^. As these nanomaterials are often subjected to external mechanical forces, understanding their mechanical properties is important. Considering the size of these nanomaterials, lateral force microscopy (LFM)^23,24^ is an effective tool for investigating the elastic moduli of nanomaterials such as ZnO^25,26^, Au^27^, Si^28^, and W nanowires^29,30^. To perform mechanical tests accurately on a single nanowire, AFM scanning is conducted along a programmed manipulation path to deflect the nanowire^25,27^. However, AFM scanning in contact mode also results in the deflection of vertically grown nanorods^26^.

Typically, LFM involves measuring the degree of torsion induced in the AFM cantilever by the surface friction using a position-sensitive photodetector (PSPD)^31–33^. However, when studying the mechanical properties of nanorods, the lateral force applied to the AFM tip may be regarded as the lateral force to which the nanorods are subjected by the AFM tip^26^. Accordingly, by simultaneously monitoring the C-AFM and LFM signals obtained while imaging vertically grown piezoelectric nanorods, the ratio of the output current to the lateral force applied to the ZnO nanorods can be determined.

We observed the variations in the C-AFM current signal in response to the application of a lateral force to ZnO nanorods by simultaneously performing C-AFM and LFM. The degree of torsion of an AFM probe can be monitored during AFM operation (including C-AFM) by integrating the AFM instrument with a scanning electron microscope^34,35^. In particular, Wen^34^ suggested that the triboelectric effect and contact potential as well as the piezoelectric effect are responsible for the current signals detected from ZnO nanorods using an AFM probe. However, this method requires a sophisticated experimental arrangement. Conversely, the C-AFM and LFM signals can be readily acquired simultaneously during AFM operation in contact mode. In this work, this novel experimental method was used to study the effect of the ZnO nanorod size on the efficiency of conversion of the mechanical force to which they are subjected into current via the piezoelectric and triboelectric effects. Note that if five ZnO nanorod samples with distinct sizes are deflected by the same AFM tip with a constant normal force, the variation in the lateral force would approximately represent the variation in the net applied mechanical force. Thus, five ZnO nanorod samples with distinct aspect ratios were prepared, and the ratio of the output current to the mechanical force applied to the nanorods was determined. With respect to AFM-based studies of the piezoelectric effect of ZnO nanorods, the choice of the AFM probe may have a determining effect on the results, because the normal force in contact mode is determined by the spring constant of the AFM probe. Therefore, the ratio of the output current to the lateral force was determined using two AFM probes with different spring constants. Finally, we attempted to elucidate the mechanism responsible for the generation of current signals in ZnO nanorods by the AFM tip based on a scatter plot of the current signal versus lateral force and pixel-wise comparison of the C-AFM and LFM images. We confirmed that the piezoelectric and triboelectric effects mainly contribute to the current signals from ZnO nanorods detected by the AFM tip. Furthermore, the contribution of the triboelectric effect is quite large when an AFM probe with a large spring constant is used with a large normal force.

## Results and discussion

### Vertically grown ZnO nanorods

**Figure 2:**
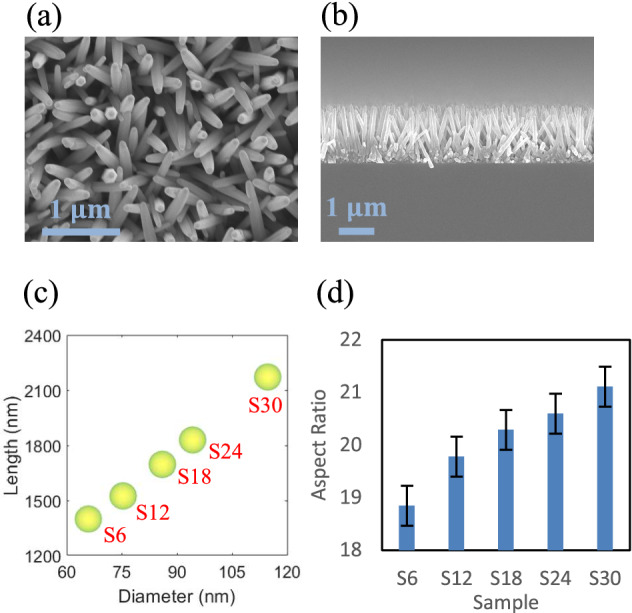
Growth of ZnO nanorod samples. (**a**) Top view and (**b**) side view of vertically grown ZnO nanorods (S18) and (**c**) lengths and diameters and (**d**) aspect ratios of five ZnO nanorod samples (S6‒S30). As the growth time increased, both the length and diameter continuously increased. (**a**) and (**b**) were obtained using the scanning electron microscope.

The lengths and diameters of the vertically grown ZnO nanorods, which were measured with a scanning electron microscopy (SEM) system, are shown in Fig. 2. Overall, the aspect ratio of the ZnO nanorods increases with increasing growth time (see Fig. 2d). The details of the nomenclature scheme used for the sample names and growth conditions are given in Methods. SEM images of the five ZnO nanorod samples are shown in Supplementary Fig. S1.

### Representations of current versus lateral force via scatter plot

**Figure 3:**
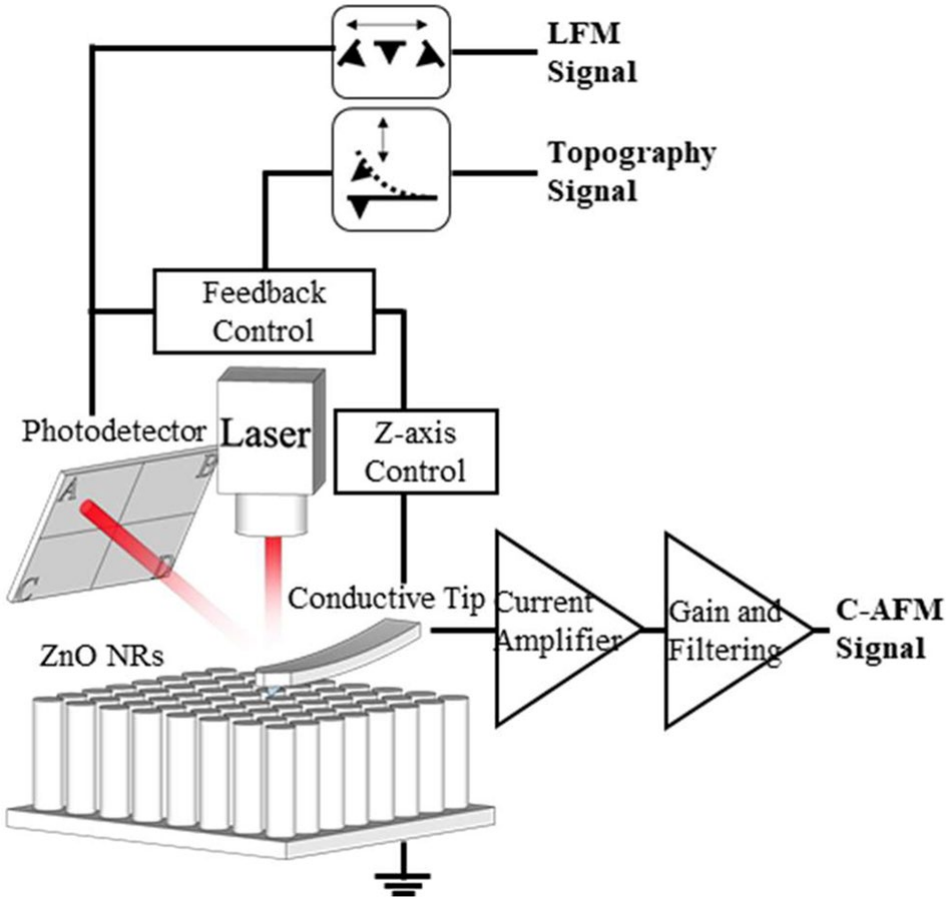
Schematic of the experimental setup. Simultaneous acquisition of current and lateral force signals during the scan of vertically grown ZnO nanorods using an AFM probe. The torsional behaviour of the AFM probe was monitored by a laser reflecting from the AFM cantilever into the position-sensitive photodetector. The current signals originating from the ZnO nanorods were detected through the AFM probe and the external circuit with the current amplifier.

**Figure 4:**
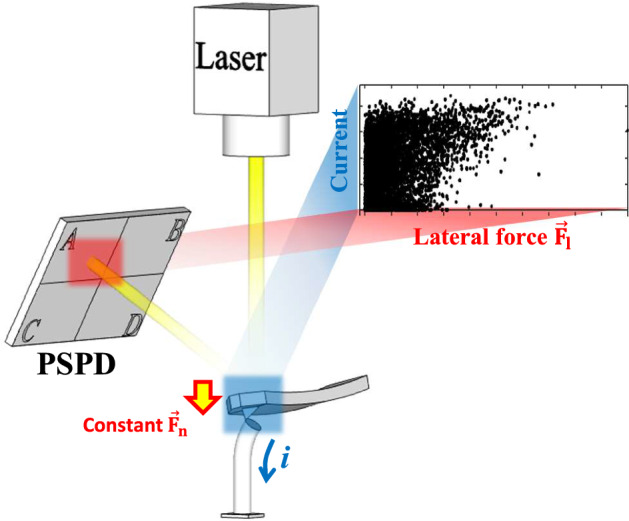
Scatter plot of current and lateral force signals. During a typical C-AFM measurement, a constant normal force was applied to the ZnO nanorods. The magnitude of the lateral force applied to the nanorods changed continuously. While completing one raster scan, two sets of C-AFM and LFM signals were simultaneously obtained from every data point on the sample surface. The scatter plot composed of these two distinct signals provides useful information for the mechanism of current generation.

As stated previously, the C-AFM and LFM signals were acquired simultaneously as an AFM probe scanned the ZnO nanorods. Whereas the LFM signal was transmitted through the PSPD, the C-AFM signal was obtained via the current amplifier (Fig. 3). Thus, there was no cross-talk between these two distinct signals. In this study, all the C-AFM and LFM measurements were obtained over a 10 µm × 5 µm area, which resulted in 512 × 128 data points. The C-AFM and LFM data points were represented as $c - afm_{ i,j}$ and $lfm_{ i,j}$, respectively, using matrix notation. As shown in Fig. 4, a scatter plot was used to represent the correlation between these two sets of data visually. The C-AFM signal from *n*-type ZnO nanorods has a negative value, whereas the LFM signal during the trace and retrace scans is positive and negative, respectively. Thus, $\left| {c - afm_{ i,j} } \right|$ and $\left| {lfm_{ i,j} } \right|$ were used for the computations and graphical representations for simplicity.

**Figure 5:**
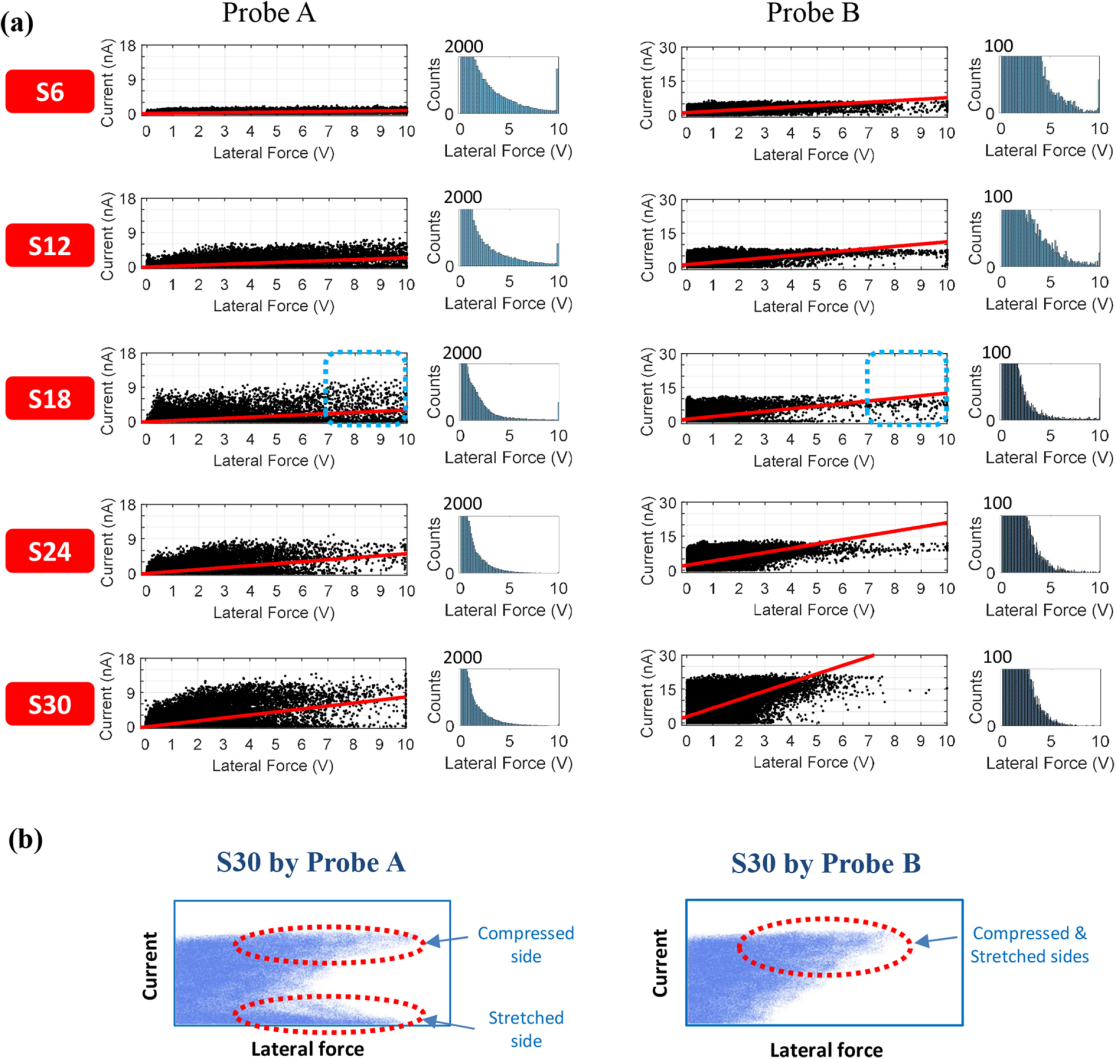
Effects of ZnO nanorod size on the ratio of the current to the lateral force. (**a**) Scatter plots of current versus lateral force signals for five samples, as measured by Probes A and B during trace scans. (**b**) Schematics illustrating the different scatter patterns between Probes A and B for S30.

The C-AFM and LFM measurements were performed on the five samples using two distinct AFM probes. The resultant scatter plots of the current versus lateral force during the trace scans are shown in Fig. 5a. Both probes (i.e., Probes A and B) were composed of Si and coated with electrically conductive materials, such as Pt, Cr, and Ir, to a thickness of less than 30 nm. The actual magnitude of the measured current is dependent on the condition of the metal layer formed on the AFM tip and its degree of wear. Therefore, it was not worth comparing the current levels of the two AFM probes. It can be seen from Fig. 5a that regardless of the type of AFM probe used, the points in the scatter plot gradually vary with increasing aspect ratio. For example, in the case of Probe B, when the aspect ratio of the ZnO nanorods is relatively small, most of the data points lie near the *x*-axis. Thus, although a moderately large lateral force was applied externally to the ZnO nanorods, only a small current was produced. As the aspect ratio increases, the pattern of the data points changes gradually. In particular, in the S30 scatter plot, the data points for lateral forces corresponding to voltages greater than 7 V are scarce. Thus, the deflection of sample S30 involved relatively smaller lateral forces than the deflection of the other samples. However, the output current from S30 is noticeably higher that of the other samples. This trend is observed in the scatter plots produced by both Probes A and B. This variation was quantitatively analyzed using the linear regression method, and the corresponding linear model is shown in each scatter plot. The curve fitting tool in Matlab was used to compute the linear model, which can be represented as

1 $$ f\left( x \right) = p_{1} x + p_{2} . $$

We also analyzed the effect of the spring constant of the AFM probe on the point pattern. In the scatter plots for S18, on comparing the number of data points for lateral forces corresponding to voltages greater than 7 V (marked by dotted boxes), it can be seen that the number of data points decreases when an AFM probe with a higher spring constant is used: Probe A (1496 data points) and Probe B (184 data points). Thus, the degree of torsion of the cantilever is reduced when an AFM probe with a larger spring constant is used to deflect the ZnO nanorods. Furthermore, in the case of Probe B, the data points for lateral forces corresponding to voltages greater than 7 V are not concentrated near the *x*-axis; in contrast to the case for Probe A.

**Figure 6:**
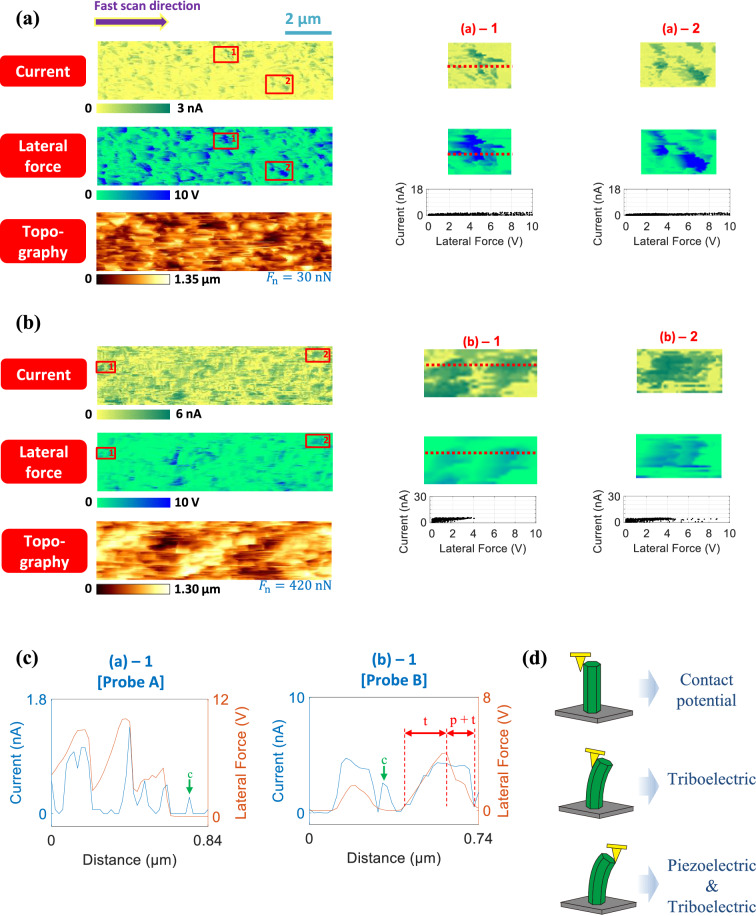
Pixel-wise comparison of the current and lateral force images. Current, lateral force, and topography images obtained using (**a**) Probe A and (**b**) Probe B during trace scans. *F*_n_ is the normal force applied to the ZnO nanorods by the AFM tip. Magnified images of the small areas marked by boxes are provided, with corresponding scatter plots on the right. (**c**) Line profiles of the magnified current and lateral force images in (**a**) and (**b**). (**d**) Schematics of three distinct current generation mechanisms: contact potential, triboelectric effect, and simultaneous piezoelectric and triboelectric effects^34^.

Such different scatter patterns obtained by Probes A and B can be depicted schematically as shown in Fig. 5b. If the generation of piezoelectric potential in an *n*-type ZnO nanorod is solely responsible for the detected current signals, the signals should be detected when an AFM tip touches the compressed side of an *n*-type ZnO nanorod^9^. If so, the scatter pattern should be similar to the schematic called “S30 by Probe A.” However, in the scatter pattern obtained by Probe B, the data points originating from the stretched side are not observed near the lateral force axis. This finding implies that the generation of piezoelectric potential is not only mechanism responsible for the detected current signals. Similar characteristics are also observed in the set of scatter plots corresponding to the retrace scans, as shown in Supplementary Fig. S2. The mechanism underlying the detected current signals from ZnO nanorods will be elucidated in Fig. 6.

### Interpretation of scatter plots based on pixel-wise comparison of current and lateral force images

Although the scatter plots in Fig. 5 present a novel method for assessing the conversion efficiency, they do not intuitively explain the interactions between the AFM tip and ZnO nanorods. Consequently, we performed local analysis based on pixel-wise comparison of the current and lateral force images. The two pairs of C-AFM and LFM images with 512 × 128 data points corresponding to sample S6, which were obtained during the trace scan using Probes A and B, are shown in Fig. 6a and b, respectively. (See Supplementary Fig. S3 for the images obtained during the retrace scan.) It should be noted that the C-AFM and LFM images acquired during the trace scans (Fig. 6a and b) were, to some extent, different from those obtained during the retrace scans. This discrepancy could be justified by two reasons. Firstly, the ZnO nanorods were originally deflected during the growth process, as can be seen from Supplementary Fig. S1. Thus, the contacting processes between the AFM tip and nanorods would be different depending on the scan directions. Secondly, it might take finite time for the nanorods, deflected by the AFM tip, to recover their initial positions. Each C-AFM and LFM image is marked by rectangles “1” and “2,” and magnified images corresponding to these marked areas are shown on the right.

It can be seen from Fig. 6 that there is a strong correlation between the C-AFM and LFM images. Furthermore, the degree of correlation is significantly influenced by the type of AFM probe used. On directly comparing the LFM images in Fig. 6a and b, it can be seen that the degree of torsion in the AFM probe is lower for the probe with the larger spring constant (i.e., Probe B). For each pair of local C-AFM and LFM images, the scatter plot of the current versus the lateral force is displayed below the images. These scatter plots of the local images resemble the scatter plot for the complete images in Fig. 5. Furthermore, the proximity of the data points to the *x*-axis was determined by the spring constant of the AFM probe used, as is evident from comparison of the local C-AFM and LFM images. For example, when Probe A was used, some active areas in the LFM image ((a)-1 in Fig. 6a) do not correspond to the active areas in the C-AFM image. This discrepancy exists because when Probe A, which had a spring constant of 0.2 N/m, was used, torsion was occasionally induced in the AFM probe without the deflection of the ZnO nanorods. Thus, numerous data points lie near the *x*-axis. In contrast, even a small degree of torsion in Probe B, which had a spring constant of 42 N/m, resulted in the deflection of the ZnO nanorods and the generation of a current signal. Hence, numerous data points lie some distance from the *x*-axis.

However, the difference in the scatter patterns between Probes A and B shown in Fig. 5b is not fully explained yet. Thus, we examined the current and lateral force signals in Fig. 6a and b more closely. As mentioned earlier, it is known that a current signal is detected when an AFM tip touches the compressed side of an *n*-type ZnO nanorod^9^. This established theory is clearly supported by line profile (a)-1 in Fig. 6c, which corresponds to Probe A. However, when Probe B was used for imaging, a considerable amount of current signals was detected when the AFM tip was in contact with the stretched side of the ZnO nanorod (see line profile (b)-1 in Fig. 6c). This result is obviously inconsistent with the previously established theory^9^. Recently, it was reported that the current signals detected by an AFM probe originate from the triboelectric effect as well as the piezoelectric effect^34^. Thus, the current signals in line profile (b)-1 can be classified into the region in which the triboelectric effect is dominant (marked by “t”) and the region in which both triboelectric and piezoelectric effects simultaneously contribute (marked by “p + t”). The current signals contributed by the triboelectric effect are remarkable when the normal force of 420 nN was applied by Probe B. In contrast, the current signals induced by the triboelectric effect are scarcely noticeable in line profile (a)-1 due to the small amount of normal force by Probe A^36^. The different scatter patterns in Fig. 5b are now well explained. It should be noted that the proportion of current signals originating from the triboelectric effect could not be quantitatively identified from the experimental data since both piezoelectric and triboelectric effects simultaneously occurred in the ‘p + t’ region. Additionally, current signals were detected even after the LFM signals had diminished, as marked by the arrows (“c”) in line profiles (a)-1 and (b)-1. This finding implies that current was generated even after the torsion in the probe had been released. It is speculated that these current signals are generated by the contact potential between the AFM probe and ZnO nanorods^34^.

**Figure 7:**
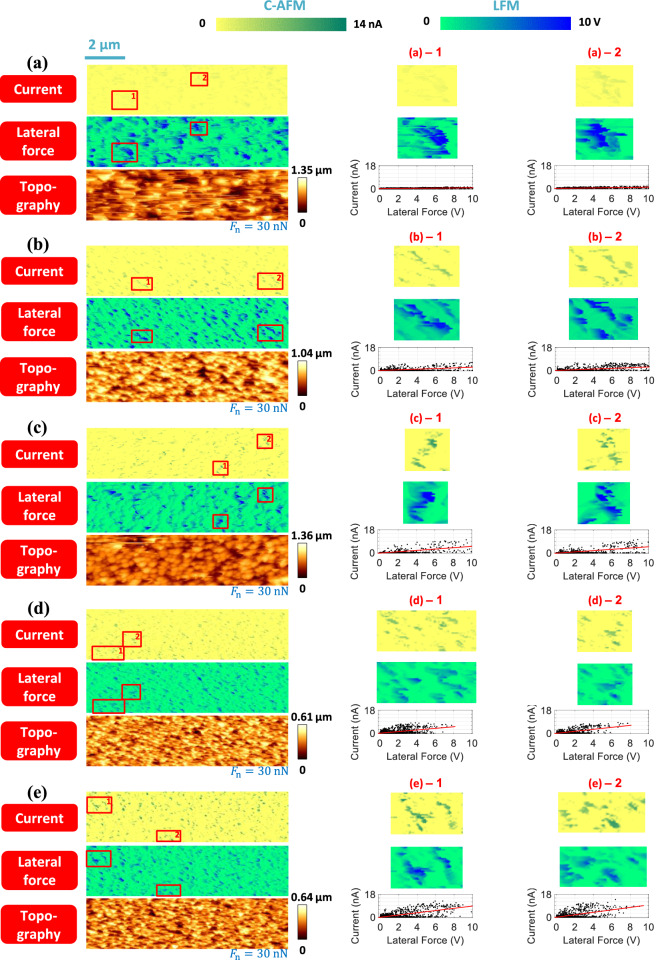
Variations in current and lateral force signals with the size of the ZnO nanorods. Current, lateral force, and topography images for (**a**) S6, (**b**) S12, (**c**) S18, (**d**) S24, and (**e**) S30 as measured by Probe A. *F*_n_ is the normal force applied to the ZnO nanorods by the AFM tip. In each figure part, magnified images of the areas marked by boxes and corresponding scatter plots are shown on the right. All images were obtained during trace scans.

The variation in the scatter plots for the current versus the lateral force with respect to the aspect ratio of the ZnO nanorods was obtained from Fig. 5. The gradual variation in the scatter plot in the case of Probe A is well explained by the results of the pixel-wise comparison of the C-AFM and LFM images, as shown in Fig. 7. All the C-AFM and LFM images were generated based on the color bars shown on the top in Fig. 7. Hence, all variations in the C-AFM and LFM signals with changes in the aspect ratio of the ZnO nanorods could be observed. It can be seen from the magnified images and their corresponding scatter plots that the current increases and the lateral force decreases as the aspect ratio increases. The same analysis results for Probe B can be found from Supplementary Fig. S4.

### Conversion efficiency of applied mechanical force into current via piezoelectric and triboelectric effects

As previously mentioned, when C-AFM and LFM are performed on ZnO nanorods, the net external force comprises the normal and lateral forces. For each AFM probe, the measurements were obtained in contact mode under a constant normal force corresponding to the setpoint of the feedback circuit. This approach allowed for the following approximation of the conversion efficiency:

2 $$ \frac{{\text{Total output current}}}{{\text{Total input mechanical force}}} \approx \alpha \frac{{\mathop \sum \nolimits_{i = 1, j = 1}^{i = 128, j = 512} \left| {c - afm_{ i,j} } \right|}}{{\mathop \sum \nolimits_{i = 1, j = 1}^{i = 128, j = 512} \left| {lfm_{ i,j} } \right|}}, $$

where $\alpha$ is a scaling factor that considers the setpoint level and electrical conductivity of the AFM tip. The effect of $\alpha$ on the conversion efficiency became almost negligible when the same AFM probe was used for all the five samples (S6‒S30) along with a constant setpoint. Note that there was no noticeable wear of the AFM tip during the measurements of the five samples. Therefore, the conversion efficiency was computed for the five samples for each AFM probe during both the trace and retrace scans.

**Figure 8:**
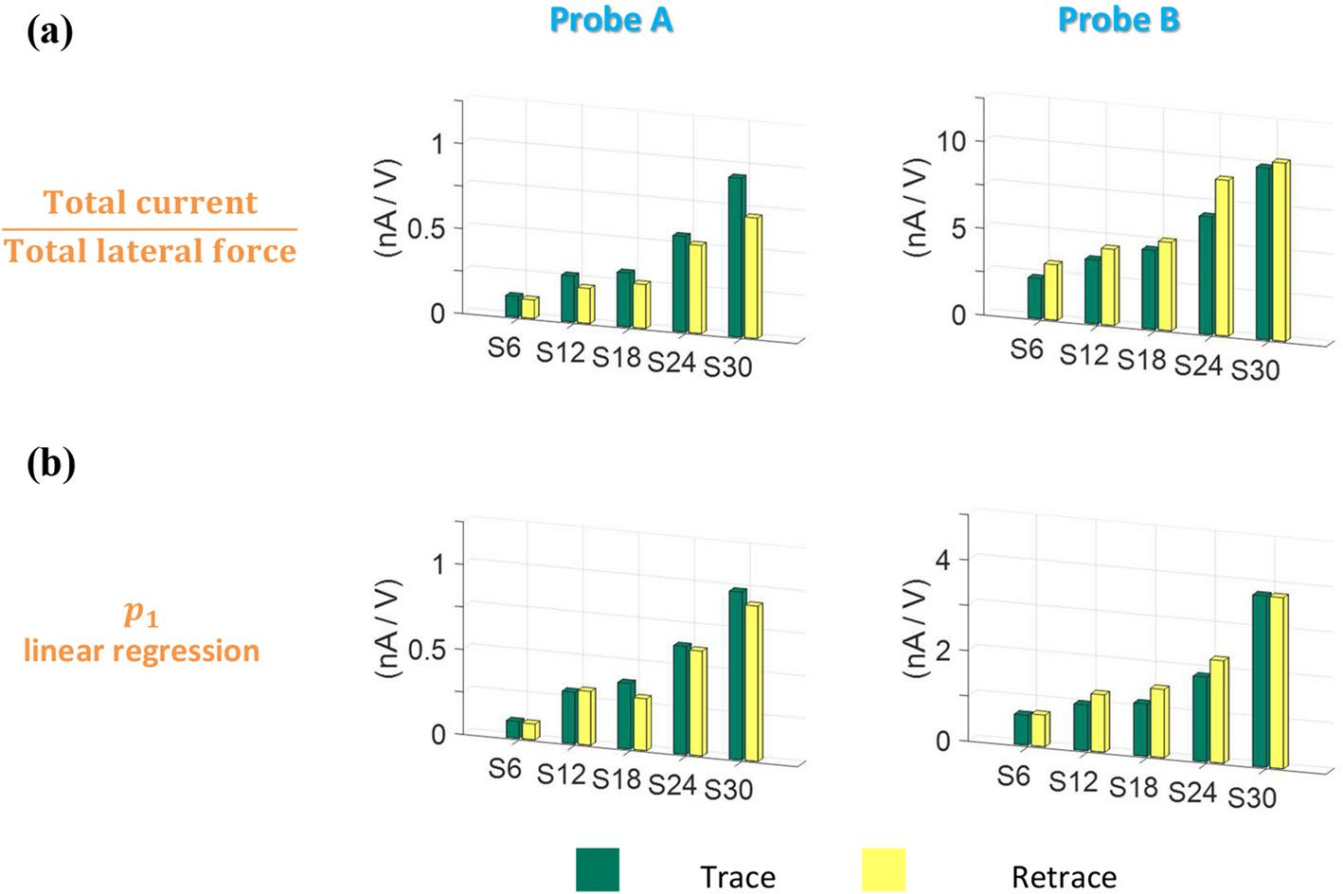
Efficiency of conversion of the lateral force applied to the ZnO nanorods into current depending on the ZnO nanorod size. (**a**) Conversion efficiencies (total current/total lateral force) computed for each sample during trace and retrace scans using Probes A and B. (**b**) *p*_1_ values for linear models Eq. (1) computed for each sample during trace and retrace scans by Probes A and B.

As shown in Fig. 8a, the conversion efficiency computed from the trace and retrace scan data sets increases with increasing aspect ratio. The conversion efficiency is, simply put, the ratio of the total current to the total lateral force. Thus, in the cases in which the data points are concentrated near the *y*-axis, the conversion efficiency is large. The pattern of the data points could be effectively described by the slope of the linear model (*p*_1_) in Eq. (1). The computed *p*_1_ values for the five samples and two AFM probes are shown in Fig. 8b. Overall, *p*_1_ also increases as the aspect ratio increases. The results in Fig. 8b suggest that a larger amount of current is generated by the ZnO nanorods for a smaller external mechanical force as the aspect ratio of the nanorods increases. Therefore, the results of the linear regression analysis agree with those of the conversion efficiency calculations performed using Eq. (2).

As can be seen in Fig. 6c, the current signals originating from the triboelectric effect are significant when a 420 nN normal force is applied by Probe B. The measured current signals contributed by the triboelectric effect are effectively reduced with a 30 nN normal force by Probe A. Generally, triboelectricity can be enhanced by increasing the normal force with respect to the sliding surface^37^, and the contact force (that is, set point) applied to the ZnO nanorods by the AFM tip remains constant by the feedback circuit. Thus, the amount of triboelectricity generated during the deflection of nanorods by the AFM tip should increase as a lateral force signal increases. The total lateral force, $l_{{{\text{tot}}}} \left( { = \sum\nolimits_{i = 1, j = 1}^{i = 128, j = 512} {\left| {lfm_{ i,j} } \right|} } \right)$, from Probe A was computed for the five samples and was found to decrease with increasing aspect ratio (Supplementary Fig. S5). This finding implies that, for Probe A, the increased conversion efficiency with increasing aspect ratio in Fig. 8a is not induced by enhanced triboelectric effect. Therefore, for Probe A, the enhanced piezoelectric potential with increasing aspect ratio of the ZnO nanorod is responsible for the increased conversion efficiency in Fig. 8a and the increase in *p*_1_ in Fig. 8b. Likewise, *l*_tot_ for Probe B does not show any increasing or decreasing trend with increasing aspect ratio of the ZnO nanorod. Thus, it can be concluded that although the effects of triboelectricity on the measured current signals are considerable, the increases in the current signals with increasing aspect ratio were not induced by triboelectric effect.

The experimental results shown in Figs. 5 and 8 for Probe A agree with those of previous works on the effect of the ZnO nanorod size on piezoelectricity^11,38,39^. It has been reported that the electrical energy increases as the aspect ratio of a ZnO nanorod increases through finite element simulations^11,39^ and experimental work^38^. This behavior was explained by two factors. First, the amount of deflection increases with increasing aspect ratio of the ZnO nanorod. Second, the free carrier concentration induced by crystallographic defects decreases as the aspect ratio increases, enhancing the piezoelectric effect. In the present study, the output current increased and the actual mechanical force deflecting the ZnO nanorods decreased as the aspect ratio of the ZnO nanorods increased. The increase in current with the increasing growth time of the ZnO nanorods was also attributed to the lower density of nanorods. Generally, when the density of the nanorods is large, a nanorod cluster is likely to form, and nanorod-to-nanorod collision takes place. As per Pan et al.^40^, the current volume generated by each nanorod reduced when a nanorod cluster was formed. As can be seen from Supplementary Fig. S1, the density of the nanorods decreased with increasing growth time. Therefore, it was speculated that the increase in current with respect to the growth time was influenced by the reduced density of nanorods, as well as the enhanced piezoelectric potential by the increased aspect ratio. Nanorod clustering can be suppressed by coating the nanorods with a flexible polymer (e.g., PMMA) and subsequently, etching with oxygen plasma^40^.

In general, the sizes and structures of freestanding piezoelectric nanomaterials are determined by the growth method and conditions used. The experimental and analysis methods proposed in this study enable one to assess readily the efficiency of conversion of the mechanical force applied to nanomaterials into current. Thus, these methods should facilitate the optimization of the synthesis of nanomaterials for use in piezoelectric sensors and energy harvesters. Additionally, it is important to use an AFM probe with a small spring constant to minimize the influence of the triboelectric effect on the results.

## Conclusions

In this study, we performed comprehensive analysis of the piezoelectric and triboelectric effects generated in ZnO nanorods using an AFM probe. By simultaneously acquiring the C-AFM and LFM signals under a constant normal force, the variations in the ratio of the output current to the applied mechanical force with changes in the aspect ratio of the ZnO nanorods could be observed. In particular, two AFM probes with spring constants of 0.2 and 42.0 N/m were used to investigate the effects of the spring constant on the analysis results. The efficiency of the conversion of the lateral force into the output current was computed, and it was found that the efficiency was enhanced with increasing aspect ratio of the ZnO nanorods. In addition, scatter plots of the current versus the lateral force were produced, and the variations in the patterns of the data points with changes in the aspect ratio were analyzed quantitatively using linear regression. The resulting linear model showed that the slope increased with increasing aspect ratio. This finding indicates that there is a transition of the data points in the scatter plots from the lateral force axis (*x*-axis) towards the current axis (*y*-axis) as the aspect ratio increases. The variations in the scatter patterns with changes in the aspect ratio of the ZnO nanorods agreed with the results of a pixel-wise comparison of the C-AFM and LFM images. It was confirmed that the current signals were contributed by both the piezoelectric and triboelectric effects in a ZnO nanorod. The effect of triboelectricity on the measured current signals was alleviated by reducing a normal force and the spring constant of an AFM probe. This analysis method is expected to be applicable to all freestanding piezoelectric nanomaterials. Furthermore, it is anticipated to facilitate analysis of the effects of the growth conditions of nanomaterials on the efficiency of conversion of the applied mechanical force into the output current.

## Methods

### Growth of ZnO nanorods

The ZnO nanorods used in this study were synthesized on *p*-Si (100) substrates via a two-step hydrothermal method^41,42^. The Si substrates were sonicated in acetone, ethanol, and deionized water for 10 min each. The seed layer was grown in 50 mM zinc acetate dihydrate (Zn(Ch_3_COO)_2_·2H_2_O), dissolved in ethanol at 80 °C for 30 s, and then dried on a hot plate at 100 °C for 5 min. This process was repeated three times. The ZnO nanorods were grown on top of the seed layer using a precursor solution that was a mixture of 50 mM zinc nitrate hexahydrate (Zn(NO_3_)_2_·6H_2_O) and hexamethylenetetramine ((CH_2_)_6_N_4_) dissolved in deionized water at 80 °C. Five nanorod samples with distinct aspect ratios were prepared by varying the growth time (Table 1). The lengths and diameters of the ZnO nanorods were measured from their field emission scanning electron microscopy (JSM6700F, JEOL) images.

**Table 1:** Growth conditions for ZnO nanorod samples.

Sample name	Precursor concentration	Temperature	Growth time (h)
S6	50 mM	80 °C	6
S12			12
S18			18
S24			24
S30			30

### Conductive atomic force microscopy and lateral force microscopy

The C-AFM and LFM measurements were obtained using an AFM instrument (XE-150, Park Systems). A C-AFM module with a current amplifier was installed in the AFM system. In this work, two types of AFM probes were used, as listed in Table 2. The spring constants of these AFM probes were 0.2 and 42.0 N/m. The appropriate set point for each AFM probe is approximately 10 times the spring constant for stable AFM operation in contact mode. However, the spring constant of Probe A was too small to deflect the ZnO nanorods, and, therefore, a set point of 30 nN was applied. As reported in the previous study by Lanza et al.^43^, current maps were significantly influenced by the set point (contact force). Thus, in the present work, the appropriate set points for Probe A and B were selected so that ZnO nanorods could be sufficiently deflected while ensuring that the AFM tip does not wear out rapidly. Table 2 lists the conditions for the AFM scans performed using the two probes. During all the AFM measurements, the cantilever was consistently oriented perpendicular to the fast-scan direction.

**Table 2:** AFM scan conditions for the two AFM probes used.

	AFM probes	Spring constant (N/m)	Set point (nN)	Scan speed (Hz)
Probe A	ElectriCont-G	0.2	30	0.2
Probe B	PPP-NCHPt	42.0	420	0.2

## Supplementary Information

Supplementary Information.

## Data Availability

The datasets generated during and/or analysed during the current study are available from the corresponding author on reasonable request.
