# Supplementary material for: Simultaneous acquisition of current and lateral force signals during AFM for characterising the piezoelectric and triboelectric effects of ZnO nanorods
Source: Sci Rep. 2021 Feb 3;11:2904. doi: 10.1038/s41598-021-82506-8 (PMC7859228; doi:10.1038/s41598-021-82506-8)
Supplement: Supplementary file 1 — Supplementary Information. [file 41598_2021_82506_MOESM1_ESM.pdf]

Supplementary Information: Simultaneous acquisition of current and lateral force signals during AFM for characterising the piezoelectric and triboelectric effects of ZnO nanorods

Yijun Yang [1], Kwanlae Kim [1,*]

[1] Department of Manufacturing Systems and Design Engineering (MSDE), Seoul National University of Science and Technology (SeoulTech), Seoul 01811, Republic of Korea

[*] klkim@seoultech.ac.kr

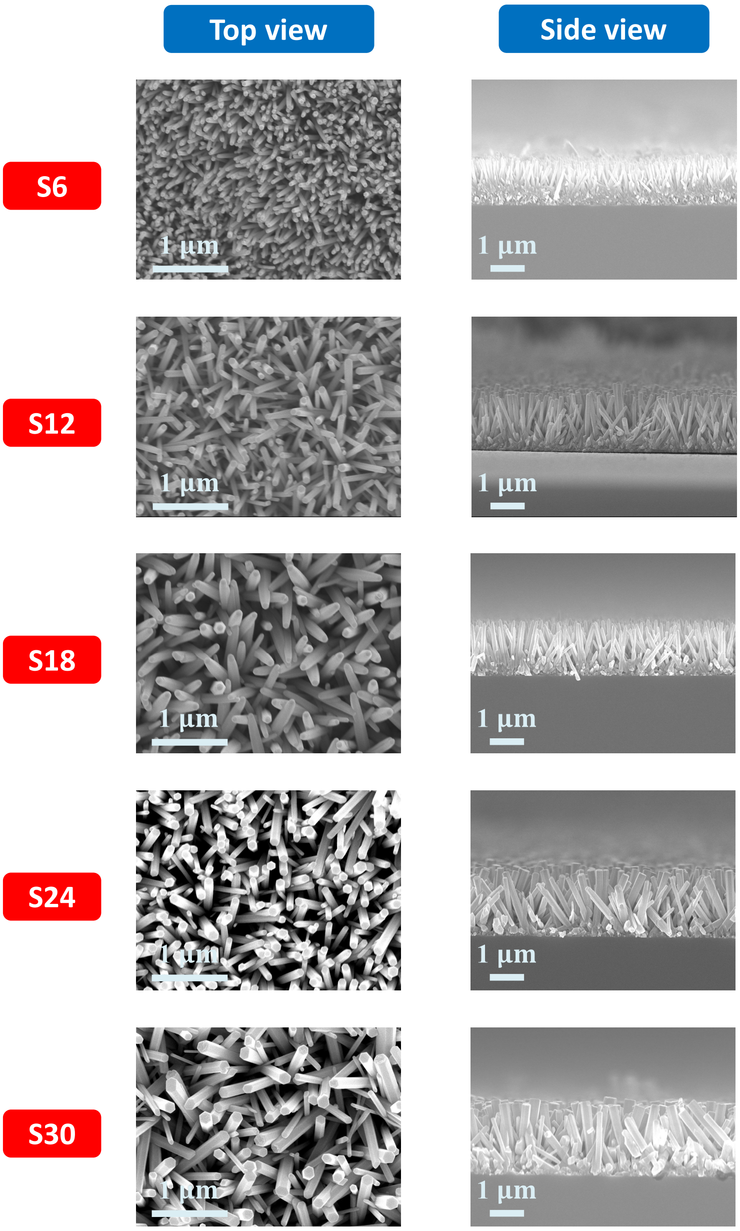

Supplementary Figure S1. Observation of the five ZnO nanorod samples. The top and side views of vertically grown ZnO nanorods. The images were taken by a scanning electron microscope.

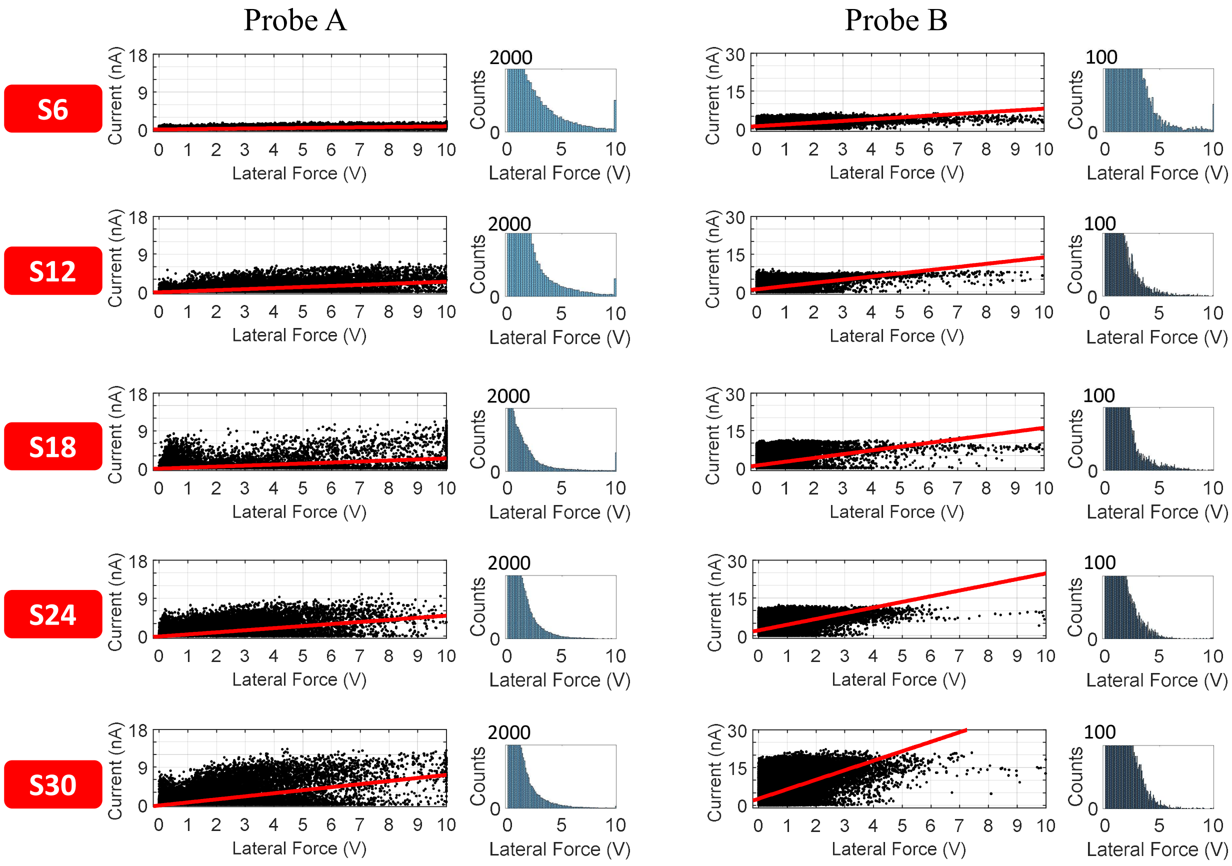

Supplementary Figure S2. Scatter plots of current versus lateral force for the five samples measured by Probes A and B during retrace scans by simultaneous acquisition of the current and lateral force signals.

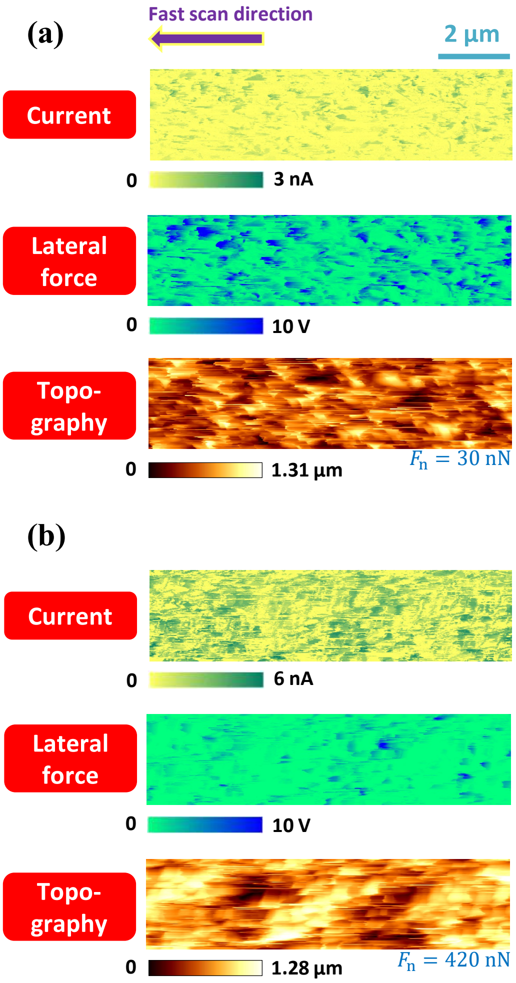

Supplementary Figure S3. Current, lateral force, and topography images obtained by (a) Probe A, and (b) Probe B during retrace scans. (a) and (b) were taken from the same areas in Figs. 6(a) and 6(b), respectively. F_n is the normal force applied to the ZnO nanorods by the AFM tip.

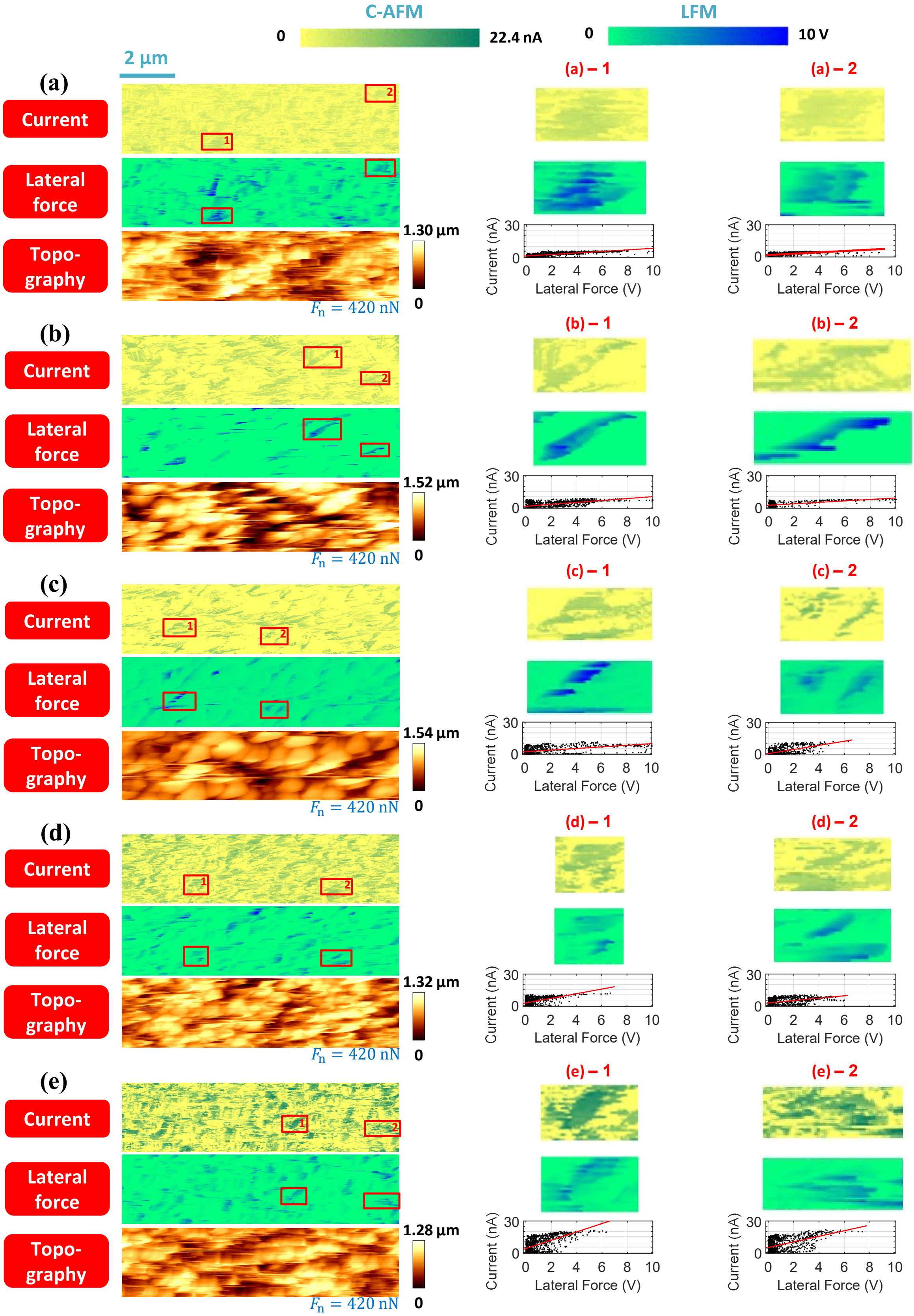

Supplementary Figure S4. Variations of current and lateral force signals with ZnO nanorod size. Current, lateral force, and topography images for (a) S6, (b) S12, (c) S18, (d) S24, and (e) S30, as measured by Probe B. In each image, small areas are marked by boxes, and their enlarged images and scatter plots are shown on the right side. F_n is the normal force applied to the ZnO nanorods by the AFM tip. All these images were taken during trace scans.

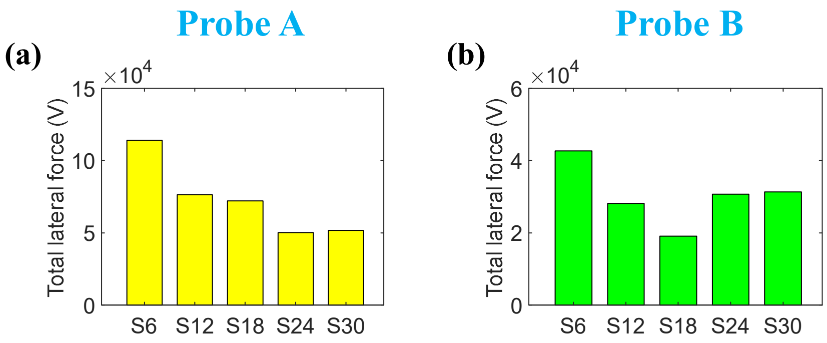

Supplementary Figure S5. Sum of lateral force signals for samples S6–S30 acquired by Probes A and B. The computation was conducted using the data in Fig. 5. For each sample, the total lateral force was computed using $\sum_{i=1,j=1}^{i=128,j=512} \left| lfm_{i,j} \right|$.
